# The UCSC Cancer Genomics Browser: update 2015

**DOI:** 10.1093/nar/gku1073

**Published:** 2014-11-11

**Authors:** Mary Goldman, Brian Craft, Teresa Swatloski, Melissa Cline, Olena Morozova, Mark Diekhans, David Haussler, Jingchun Zhu

**Affiliations:** 1 Center for Biomolecular Science and Engineering, University of California at Santa Cruz, Santa Cruz, CA 95064, USA; 2 Howard Hughes Medical Institute, University of California at Santa Cruz, Santa Cruz, CA, USA

## Abstract

The UCSC Cancer Genomics Browser (https://genome-cancer.ucsc.edu/) is a web-based application that integrates relevant data, analysis and visualization, allowing users to easily discover and share their research observations. Users can explore the relationship between genomic alterations and phenotypes by visualizing various -omic data alongside clinical and phenotypic features, such as age, subtype classifications and genomic biomarkers. The Cancer Genomics Browser currently hosts 575 public datasets from genome-wide analyses of over 227 000 samples, including datasets from TCGA, CCLE, Connectivity Map and TARGET. Users can download and upload clinical data, generate Kaplan–Meier plots dynamically, export data directly to Galaxy for analysis, plus generate URL bookmarks of specific views of the data to share with others.

## INTRODUCTION

Cancer is a genomic disease that results in uncontrolled cell growth (1). To decode this disease, many large-scale genome characterization efforts have worked to generate and interpret data at an unprecedented scale. For example, The Cancer Genome Atlas (TCGA) project already has close to a petabyte of next-generation sequencing data with over 20 000 samples (https://cghub.ucsc.edu, http://cancergenome.nih.gov/) (2–4) and is continuing to grow. In addition to these efforts, many researchers are generating large-scale cancer genomics datasets for their own investigations. Transforming these new data into knowledge requires effective, user-friendly visualization and computational tools that can be used by both clinical researchers and informaticians (5).

The UCSC Cancer Genomics Browser (https://genome-cancer.ucsc.edu/) is a web-based application that was developed in response to an urgent demand in the cancer research field for integrative visualization of large, complex genomic datasets arising from different technology platforms. Our browser integrates relevant data with analysis and visualization tools, allowing users to easily discover, analyze and share relevant research. It displays whole-genome and sets-of-genes views of genome-wide experimental measurements for sets of samples alongside their associated phenotype information. Researchers can explore the relationship between genomic alterations and phenotypes by visualizing various genomic data alongside clinical and phenotypic features, such as age, subtype classifications and genomic biomarkers. Viewing data from multiple experiments on the same samples side-by-side allows users to make inferences across different data types. Integrated Kaplan–Meier survival analysis helps investigators assess survival stratification by any type of information, including clinical features, genomic annotations and user-defined subgroups and signatures. User accounts allow saving of bookmarks, relevant sets of genes and interesting genomic signatures. These bookmarks take the form of URLs, which can be easily shared.

The Cancer Genomics Browser currently hosts 575 open-access datasets from genome-wide analyses of over 227 000 samples, including 526 datasets from 31 different TCGA cancer types. Types of hosted datasets include copy number, somatic mutation, DNA methylation, gene and exon expression, protein expression, PARADIGM pathway inference (6) and phenotype data. Our automated pipeline updates TCGA data periodically, ensuring we are visualizing the most recent data available. Additionally, our pipeline ingests TCGA phenotype data and attempts to assign more readable feature names and values. We further derive overall and recurrence free survival from TCGA phenotype data, allowing users to perform survival analysis.

We also host data from CCLE (Cancer Cell Line Encyclopedia), which profiles 1000 cell lines and their responses to 24 drugs; Connectivity Map, which generates expression profiles of several cells lines when exposed to hundreds of different small molecules (7); and childhood cancers (8–10). We also host a number of protected datasets including pre-publication data. A control mechanism restricts the access of private data to authorized users.

We have made significant improvements to the browser in the past 2 years. Users now can download and upload phenotype data, generate Kaplan–Meier plots dynamically, export data directly into Galaxy and bookmark interesting views of the data for themselves or to share with others. New data include gene-level somatic mutation and Pan-Cancer data (11), both from TCGA, and data from TARGET (Therapeutically Applicable Research to Generate Effective Treatments, https://ocg.cancer.gov/programs/target) (9) and Connectivity Map (7).

## NEW FEATURES

### Dataset search and map index

It is now possible to search and sort datasets with our new Dataset Viewer, giving users easier access to information of interest and generally offering a more complete view of the available data. For example, users can search for ‘TCGA gene expression’ or ‘pan-cancer’ datasets using a plain text interface. Our Map Index to the left of the main heatmap area shows which datasets are open, allowing users to quickly view which datasets are already displayed or jump to another dataset. Both of these features make the data in our repository more transparent to the user.

### Download and upload phenotype data

We already offer downloads of entire datasets, but these files can be difficult to manipulate due to their large size. Alternatively, users can now download just the phenotype data in view. This allows users to easily download the few clinical and genomic (using the signature feature) columns of interest for analysis and offline use. Our download format can easily be opened by most spreadsheet-like applications and is also recognized by more advanced analysis programs, such as R.

**Figure 1.**
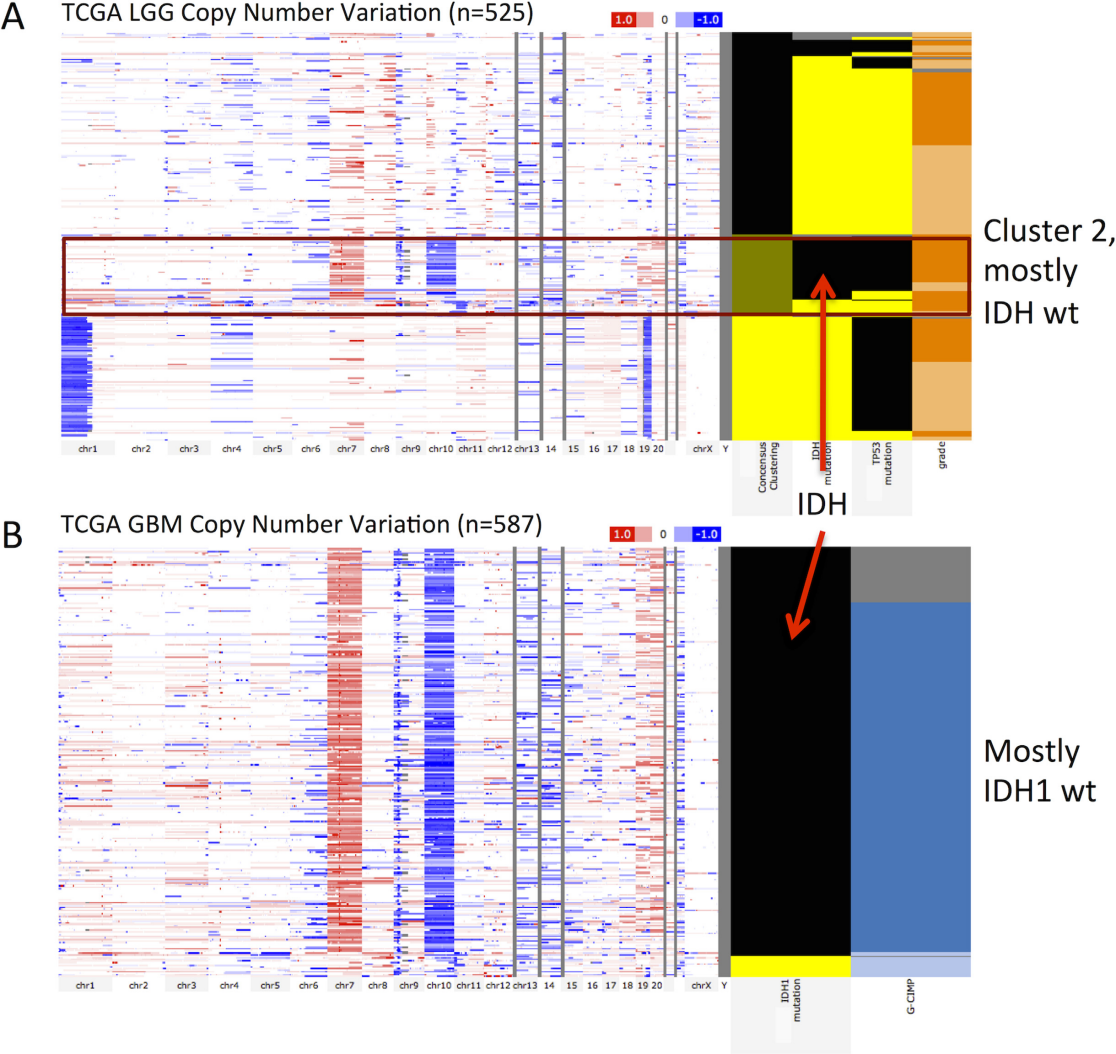
TCGA LGG (*n* = 525) and GBM genomics (*n* = 587) datasets showing a common molecular subtype of similar copy number variation profile for both LGG (red box, panel **A**) and GBM IDH wild-type patients (panel **B**). In each panel the genomic heatmap is on the left and the clinical heatmap is on the right. Copy number datasets use red and blue to represent amplification and deletion, respectively. Black color for the IDH mutation feature indicates wild-type IDH. For all columns showing mutation status, yellow indicates that a non-silent somatic mutation (nonsense, missense, frame-shift indels, splice site mutations, stop codon read-throughs, change of start codon, in-frame indels) was identified in the protein-coding region of a gene and black shows that none of these previous mutation calls were identified. Gray represents no data. A bookmark of this view is at https://genome-cancer.ucsc.edu/proj/site/hgHeatmap/#?bookmark=ff9e8550141e6f37e3ec242152066914 (A) TCGA LGG whole-genome copy number variation. Left-most column in the clinical heatmap shows the consensus clustering assignment with Cluster 1 as yellow, Cluster 2 as green and Cluster 3 as black. Note that Cluster 2 is mostly IDH wild type. The next column shows IDH1 or IDH2 mutants and third column shows TP53 mutation. The last column shows tumor grade with light orange being grade 2 and dark orange being grade 3. (B) TCGA GBM whole-genome copy number variation. Left-most column in the clinical heatmap shows IDH1 mutation status. Unlike the LGG cohort, the GBM cohort harbors mutations in IDH1 and not in IDH2. The second column shows the glioma-CpG island methylator phenotype (G-CIMP) with light blue representing G-CIMP tumors and dark blue indicating that it is not characterized as a G-CIMP tumor.

**Figure 2.**
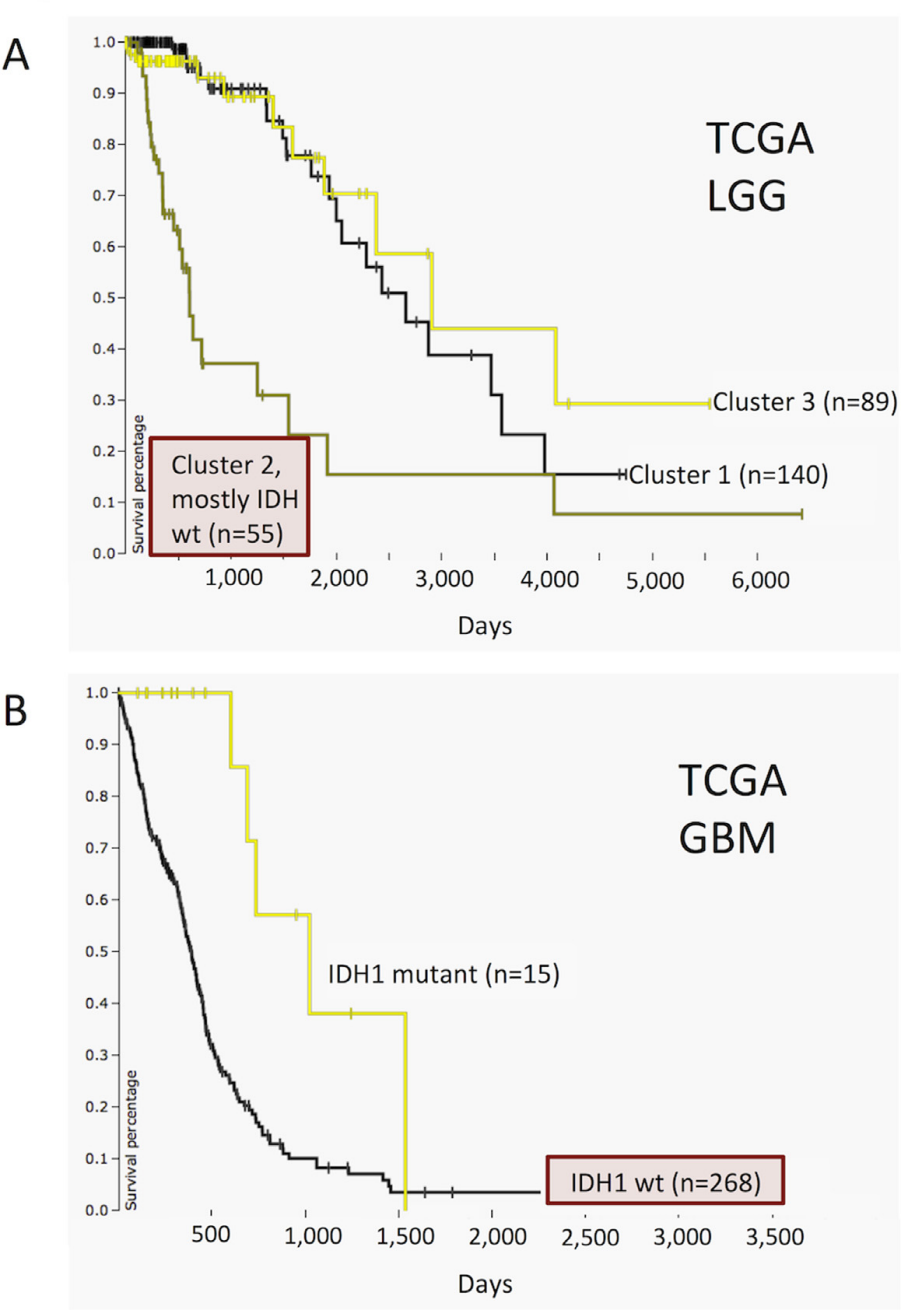
TCGA LGG and GBM datasets showing differential survival. It demonstrates that IDH wild-type subtypes in both cancers have worse prognosis compared to the rest of the tumors of the same cancer type. Time (X-axis) for both panels is in days. (**A**) Kaplan–Meier plot for TCGA LGG cohort. Patients grouped by consensus clustering assignment with Cluster 1 as yellow, Cluster 2 (mostly IDH wild type) as green and Cluster 3 as black. (**B**) Kaplan–Meier plot for TCGA GBM cohort. Patients clustered by IDH1 mutation status with yellow indicating that a non-silent somatic mutation (nonsense, missense, frame-shift indels, splice site mutations, stop codon read-throughs, change of start codon, in-frame indels) was identified in the protein-coding region of a gene and black indicating that none of these mutations were identified.

Users can now upload any numerical data to the clinical heatmap, allowing visualization of their own annotations. In conjunction with phenotype data download, this facilitates powerful, iterative cycles of analysis and visualization. Users download data they are interested in, manipulate or analyze it (e.g. perform a clustering analysis) and then upload the results back to the browser for visualization and further analysis. This loop is also useful for viewing more than one type of genomic data side-by-side. Figure 1 shows an example of this where we downloaded TCGA Lower Grade Glioma (LGG) datasets from the browser, clustered samples by genome-wide data derived from RNA, DNA and methylation platforms, and then uploaded the clustering results back into the browser to visualize the distinct genomic characteristics of each tumor subtype (12). We identified three clusters, where Cluster 2 was enriched with IDH1 and IDH2 wild-type samples and the other two clusters were enriched for IDH1 or IDH2 mutants. Cluster 2 (mostly IDH wild type) shows a copy number variation profile (chromosome 7 amplification and chromosome 10 deletion) similar to a subtype of TCGA Glioblastoma (GBM) samples, which is also IDH1 wild type. Unlike the LGG cohort, the GBM cohort harbors mutations in IDH1 but not in IDH2. Patients in the LGG Cluster 2 (mostly IDH wild type) have a worse survival profile compared to the rest of the LGG cohort (Figure 2). Similarly to LGG, patients of IDH1 wild-type GBM subtype also tend to have poorer survival compared to the IDH1 mutant subtype. These observations suggest a common molecular subtype across two different types of brain tumors (12).

### Kaplan–Meier plots

One of our most popular features is Kaplan–Meier plots, which are visual estimates of the survival of different groups of patients over time. Percent survival is on the Y-axis and time is on the X-axis; the steeper curve, the worse the survival outcome is over time. Patients are grouped according to the clinical or genomic data of the user's choice. For continuous data, like gene expression, we automatically divide patients into three roughly equal groups. Users can quickly test many different groupings of patients to see which factors influence survival. In particular, this feature is useful in conjunction with the custom data upload, allowing users to view survival over time for any grouping of patients. Users can also download the underlying survival data, which can be used by other programs, such as R, to perform analysis offline.

### Integration with Galaxy

Integrating our browser with Galaxy, an open, web-based workflow management system for data intensive biomedical research (http://galaxyproject.org/) (13–15), enables users to do more sophisticated analysis, thanks to the wealth of contributed tools available as part of the Galaxy project. Galaxy brings sophisticated computational analyses within the reach of non-computational users by simplifying tool installation and execution, as well as providing automated provenance tracking. Within Galaxy, users can open the Cancer Browser and export data directly into Galaxy datasets. One can also upload analysis results from Galaxy into our clinical data upload, allowing instant visualization. Additionally, we have imported one of our more commonly used tools, the genome-wide *t*-test, into Galaxy. For example, this can be used to find the most differentially expressed genes between two groups of patients. Our Galaxy *t*-test tool gives users direct access to the results and also allows them to integrate our tools with others into analysis pipelines. Our tools are available in the Galaxy test toolshed.

### Bookmarks

Bookmarks allow users to save views of the data for themselves, for further analysis, or to share insights with others. Bookmarks save the state of the browser exactly as it appears, including Kaplan–Meier plots or other statistical analyses. Bookmarks take users to a live, interactive browser, allowing continued exploration of the data from the original bookmarked view. Users can create bookmarks whether or not they are logged in; however, user accounts allow users to save bookmarks for easy access.

### Help demonstrations

In addition to our user guide, FAQ and interactive tutorial, we now have example demonstrations showing users what type of analysis and visualization is possible with our browser as well as presenting some of our most popular data. We have several demonstrations ranging from basic to advanced, each consisting of a bookmark of the final analysis/visualization plus step-by-step instructions on how to reach that point.

## NEW DATA

### TCGA

We have greatly expanded our TCGA data by adding 11 new cancer types as well as numerous datasets for the cancer types we already host, for a total of 31 cancer types and 526 datasets (Table 1). Dataset types include gene-level somatic mutation calls from genomic data analysis centers (Broad Institute, Washington University, Baylor College of Medicine, University of North Carolina, BC Cancer Agency, UC Santa Cruz Genome Data Analysis Center), segmented copy number estimates generated from the Affymetrix Genome-Wide Human SNP Array 6.0 platform, gene-level copy number estimates from GISTIC2 from the TCGA FIREHOSE pipeline (http://gdac.broadinstitute.org/) (16), several gene and exon expression estimates using RNAseq and array methods, DNA methylation estimates from the Illumina Infinium HumanMethylation27 and Illumina Infinium HumanMethylation450 platforms, and phospho- and total protein expression estimates assayed by reverse phase protein array technology. We also have datasets showing integrated gene activity level inferred using the PARADIGM method (6).

**Table 1.** Dataset summary

Cancer type	DNA methylation	Pathway activity	Copy number	Gene expression	Protein expression	Somatic mutation
TCGA PANCAN12	1 (4919)		2 (9656)	1 (3599)	1 (3467)	1 (3276)
TCGA acute myeloid leukemia	2 (388)	2 (339)	4 (776)	6 (1050)		2 (393)
TCGA adrenocortical cancer	1 (80)	2 (156)	4 (360)	4 (316)	1 (46)	5 (454)
TCGA bladder urothelial carcinoma	1 (373)	2 (479)	4 (1118)	4 (1144)	2 (254)	3 (467)
TCGA brain lower grade glioma	1 (525)	4 (978)	4 (1976)	5 (2135)	1 (260)	7 (2068)
TCGA breast involve carcinoma	2 (1182)	4 (3095)	4 (4194)	5 (5325)	2 (1156)	4 (3224)
TCGA cervical & endocervical cancer	1 (260)	2 (365)	4 (876)	4 (1028)		5 (676)
TCGA colon & rectum adenocarcinoma	2 (706)	4 (1616)	2 (1178)	3 (1078)	2 (925)	1 (224)
TCGA colon adenocarcinoma	2 (527)	4 (1154)	4 (1738)	7 (1818)	2 (665)	2 (270)
TCGA diffuse large B-cell lymphoma	1 (48)	2 (56)	4 (142)	4 (112)		
TCGA esophageal carcinoma	1 (202)	2 (140)	4 (556)	2 (274)		
TCGA glioblastoma multiforme	2 (432)	4 (1336)	4 (2316)	7 (1812)	2 (429)	2 (582)
TCGA head & neck squamous cell carcinoma	1 (580)	2 (990)	4 (2046)	4 (2164)	2 (424)	2 (815)
TCGA kidney chromophobe	1 (66)	2 (132)	4 (264)	4 (364)		2 (131)
TCGA kidney clear cell carcinoma	2 (898)	4 (1170)	4 (2082)	5 (2488)	2 (908)	2 (630)
TCGA kidney papillary cell carcinoma	2 (292)	4 (427)	4 (876)	5 (1048)		6 (885)
TCGA liver hepatocellular carcinoma	1 (307)	2 (378)	4 (932)	4 (1048)		5 (1008)
TCGA lung adenocarcinoma	2 (633)	4 (1038)	4 (1978)	5 (2225)	2 (474)	2 (773)
TCGA lung cancer	2 (1197)		2 (1966)	3 (2364)	1 (432)	1 (408)
TCGA lung squamous cell carcinoma	2 (564)	4(1283)	4 (1964)	6 (2448)	2 (390)	3 (532)
TCGA mesothelioma	1 (37)		4 (148)	4 (144)		
TCGA ovarian serous cystadenocarcinoma	1 (616)	4 (1645)	4 (2322)	9 (3096)	2 (824)	5 (870)
TCGA pancreatic adenocarcinoma	1 (156)	2 (169)	4 (442)	4 (512)	1 (106)	2 (118)
TCGA pheochromocytoma & paraganglioma	1 (187)		4 (672)	4 (748)		5 (919)
TCGA Prostate adenocarcinoma	1 (385)	2 (663)	4 (1502)	4 (1884)	1 (164)	5 (1163)
TCGA rectum adenocarcinoma	2 (179)	4 (462)	4 (650)	7 (620)	2 (260)	2 (116)
TCGA sarcoma	1 (249)	2 (206)	4 (732)	4 (688)		
TCGA skin cutaneous melanoma	1 (418)		4 (1372)	4 (1544)	1 (205)	5 (1654)
TCGA stomach adenocarcinoma	2 (482)	2 (546)	4 (1516)	4 (698)	1 (268)	6 (1652)
TCGA thyroid carcinoma	1 (571)	2 (986)	4 (1998)	4 (2260)	1 (374)	3 (1156)
TCGA uterine carcinosarcoma	1 (57)	2 (113)	4 (224)	4 (228)		5 (284)
TCGA uterine corpus endometrioid carcinoma	2 (596)	4 (1153)	4 (2122)	7 (1548)	2 (604)	3 (690)
TCGA uveal melanoma	1 (80)					
Cancer Cell Line Encyclopedia			1 (972)	2 (1934)		
Connectivity map				1 (6100)		
Childhood cancer			2 (257)	4 (633)		
SU2C breast cell line			2 (92)	1 (54)		
Other datasets from literature			17 (2002)	18 (3349)		

Number of datasets by cancer type and data type; number of samples is in parenthesis.

Our newest datasets are TCGA pan-cancer data, providing researchers with a more complete cross-tumor comparison. We host all the genomic datasets published with the recent PANCAN12 paper (11), including copy number variation, gene expression, protein expression, somatic mutation, DNA methylation and subtype classifications across the 12 TCGA cancer types curated by the TCGA Pan-Cancer Analysis Working Group. These PANCAN12 datasets are under the ‘TCGA PANCAN12’ group on our interface. We have also built additional pan-cancer datasets outside the PANCAN12 paper, which are under the ‘TCGA Pan-Cancer’ group. In the second group, we have gene-level somatic mutation data for 19 cancer types, also compiled and curated by the TCGA Pan-Cancer Analysis Working Group. In addition to the efforts of the TCGA Pan-Cancer Analysis Working Group, we also have assembled gene-level copy number and gene expression across all 31 TCGA cancer types. We added pancan-normalized RNAseq data to all 31 individual cancer cohorts, allowing users to see how gene expression in a single cancer type compares to all the other TCGA cancer types. In an attempt to facilitate comparison of gene expression between TCGA and other studies, we also created gene expression datasets of percentile-ranked gene-level estimates within each sample assayed by the Illumina HiSeq platform.

### Connectivity Map

The Connectivity Map project aims to create a reference collection of gene-expression profiles of cultured human cells (MCF7, PC3, SKMEL5, HL60) treated with bioactive small molecules. With 6100 profiles over 1309 different compounds, this resource can be mined to find connections among small molecules sharing a mechanism of action, chemicals and physiological processes, and diseases and drugs (7).

### Childhood cancer

Despite the progress in treating pediatric cancers, these diseases remain a challenge to the oncologist and the long-term outcome for most high-risk pediatric cancer patients is dismal (17). While the number of pediatric cancer genomics studies is growing (https://ocg.cancer.gov/programs/target) (18,19), combined analyses of these cohorts are limited due to difficulties in data sharing and the lack of centralized analyses platforms. To support this effort we have begun to host more childhood cancer data, including 207 samples from TARGET childhood acute lymphoblastic leukemia (8), 238 samples from TARGET neuroblastoma (9), 53 samples from diffuse intrinsic pontine glioma (10) and 196 samples from NCI's Oncogenomics data repository (http://pob.abcc.ncifcrf.gov/cgi-bin/JK). We hope that our platform will become a powerful, collaborative tool for childhood cancer researchers.

### Phenotype data curation

Semantic standards are an important aspect of any informatics resource that seeks to integrate diverse datasets. The Cancer Genomics Browser has been working on this through a bottom up approach. We curated the following phenotype data elements for all the data we hosted in our database: overall and recurrence free survival information (available under phenotype data), primary disease, anatomical origin and data type such as copy number variation or somatic mutation (under dataset meta-data). The curated data enable the Kaplan–Meier survival plot functionality and also help enable the general dataset search on the front page and in the Dataset Viewer.

## FUTURE DIRECTIONS

Continuing to integrate tools and data, we are developing a new tool called Xena. Xena is a data server-based platform that stores functional genomics data and serves them in response to data requests in real-time and with minimal informatics overhead. Examples of these data requests include data visualization, data integration and further downstream analysis. The Xena data server can be installed on a laptop, servers behind a firewall, or in the cloud platform.

In conjunction we are developing the Xena Browser to access and visualize data hosted across multiple Xena servers while maintaining data privacy. The functionality allows viewing and interpretation of one's genomic data (e.g. stored on a private Xena) in the context of a large collection of cancer genomics datasets that will be stored at UCSC's Xena. In addition to data from TCGA, we look forward to integrating data from other large datasets including COSMIC (http://cancer.sanger.ac.uk/cancergenome/projects/cosmic/) (20), LINCS (http://www.lincsproject.org/) (7) and ICGC (https://icgc.org/) (21). This will be a platform for researchers to store and analyze their datasets in an interoperable manner. The current UCSC Cancer Genomics Browser and Xena Browser will coexist while we port the most popular functionalities of the Cancer Browser into the Xena Browser and develop a basic set of new functionalities. The Xena Browser will ultimately replace the UCSC Cancer Genomics Browser.

Xena is being developed to leverage the Galaxy software as the underlying workflow engine to connect with the myriad bioinformatics tools and interfaces through which Galaxy users submit private datasets for processing and analysis. The Xena cycle of visualizing, analyzing and visualizing again will provide users with a powerful tool to understand and analyze public data alongside their own. Integrating Xena as a tool module within Galaxy allows for integration and collaboration.

We will also continue to import pediatric cancer genomics datasets and make them available to the community via the Xena Browser. We have set up a special initiative termed the Treehouse Childhood Cancer Project (https://treehouse.soe.ucsc.edu) that will use the Xena Browser infrastructure and tools to serve the pediatric cancer community.
